# Outbreak of carbapenem-resistent *Acinetobacter baumannii* (CRAB) in a surgical department of a university hospital

**DOI:** 10.1186/1753-6561-5-S6-P95

**Published:** 2011-06-29

**Authors:** S Messler, K Pfeffer, R Schulze-Röbbecke

**Affiliations:** 1 Institute for Medical Microbiology and Infection Control, University of Düssesldorf, Düsseldorf, Germany

## Introduction / objectives

Multidrug resistant strains of A. baumannii are an emerging problem in hospitals. We describe the management of a CRAB outbreak including 12 cases in a surgical department comprising a 40-bed ICU and a 16-bed isolation-ward (IW) for patients with multidrug resistant organisms (MDRO). The IW was originally designed for MRSA patients only.

## Methods

Descriptive epidemiological study of the time period before and after implementation of control measures.

## Results

Between 23/1/2011 and 10/2/2011 five CRAB cases were detected in the IW. The following control measures were implemented: contact isolation and cohorting of cases, frequent disinfection of equipment and frequently touched surfaces, closure of the common bathroom and education of staff concerning contact and standard precautions. CRAB screening of all inpatients hospitalised on the ward since 15/1/2011 was performed. The screening sites were: 1) tracheal secretion or upper airway, 2) anus, perineum or groin, 3) wound. The screening revealed 3 new cases and the ward was closed for new admissions. Between 16/2/2011 and 22/2/2011 two new CRAB cases were detected on the surgical ICU and CRAB screening of all ICU patients was done revealing 2 more cases.Using repPCR, all isolates were found to be genetically identical. Further active surveillance cultures revealed no new casesuntil 15/3/2011.

## Conclusion

Consistent and immediate implementation of the infection control measures resulted in the containment of the outbreak.

## Disclosure of interest

None declared.

